# Clinical Characteristics and Long-Term Outcomes of Patients With Differing Haemoglobin Levels Undergoing Semi-Urgent and Elective Percutaneous Coronary Intervention in an Asian Population

**DOI:** 10.3389/fcvm.2022.687555

**Published:** 2022-03-18

**Authors:** Rodney Yu-Hang Soh, Ching-Hui Sia, Andie Hartanto Djohan, Rui-Huai Lau, Pei-Ying Ho, Jonathan Wen-Hui Neo, Jamie Sin-Ying Ho, Hui-Wen Sim, Tiong-Cheng Yeo, Huay-Cheem Tan, Mark Yan-Yee Chan, Joshua Ping-Yun Loh

**Affiliations:** ^1^Department of Cardiology, National University Heart Centre, Singapore, Singapore; ^2^Department of Medicine, Yong Loo Lin School of Medicine, Singapore, Singapore; ^3^School of Clinical Medicine, University of Cambridge, Cambridge, United Kingdom

**Keywords:** anaemia, percutaneous coronary intervention, Asian, bleeding event, cardiovascular outcomes

## Abstract

**Introduction:**

This study aimed to investigate the impact of anaemia on long-term clinical outcomes in patients who underwent semi-urgent and elective percutaneous coronary intervention (PCI) in an Asian population. Although the effects of anaemia on outcomes in Asian patients are well studied for acute coronary syndrome, its impact on Asian patients undergoing semi-urgent and elective PCI is unclear.

**Methods:**

This was a retrospective cohort study of patients who underwent semi-urgent and elective PCI from January 1, 2014, to December 31, 2015, at a tertiary academic centre. A total of 1,685 patients were included. They were stratified into three groups: normal (≥12 g/dL), intermediate (10–11.9 g/dL), and low (<10 g/dL) haemoglobin levels. Demographics, risk factors, and end-points including the 5-point major adverse cardiac and cerebrovascular events (MACCE) (all-cause death, subsequent stroke, myocardial infarction, congestive cardiac failure, and target lesion revascularisation), cardiovascular death, and bleeding events were analysed.

**Results:**

Patients in intermediate and low haemoglobin level groups were older with more comorbidities. Compared to the normal haemoglobin level group, low haemoglobin level group patients were associated with an increased risk of composite endpoints of all-cause death, subsequent stroke, myocardial infarction, congestive cardiac failure, and target lesion revascularisation [adjusted hazard ratio (aHR) 1.89, 95% confidence interval (CI):1.22, 2.92; *p* = 0.004]. This was driven by the increased risk of target lesions revascularisation observed in the low haemoglobin level group compared to the normal haemoglobin level group (aHR 17.74, 95% CI: 1.74, 180.80; *p* = 0.015). The patients in the low haemoglobin level group were also associated with a higher risk of bleeding events compared to the normal haemoglobin level group (aHR 7.18, 95% CI: 1.13, 45.40; *p* = 0.036).

**Conclusion:**

In our Asian cohort, patients with anaemia undergoing PCI were associated with a higher comorbid burden. Despite adjustments for comorbidities, these patients had higher mortality and worse cardiovascular outcomes following contemporary PCI.

## Introduction

Anaemia is a common comorbidity in patients undergoing percutaneous coronary intervention (PCI), with a reported prevalence between 10 and 23% in randomised controlled trials (1). Furthermore, low haemoglobin levels were an independent risk factor for cardiovascular disease in the atherosclerosis risk in the community from the ARIC study (2, 3). Some studies have shown an association between baseline low haemoglobin levels and worse outcomes following PCI for both stable coronary artery disease and acute coronary syndromes (4–7). Conflictingly, other studies have demonstrated that low haemoglobin levels did not have a significant impact on outcomes after accounting for confounders (8, 9). These studies had limitations such as the exclusion of patients with non-ST-elevation myocardial infarction as well as patients with advanced chronic kidney disease. There was also little representation from Asian populations (4–6, 8, 9). Studies have shown differences between Asian and Caucasian patients undergoing PCI in terms of baseline, angiographic, and procedural characteristics (10–12). Therefore, to clarify this issue further, our study aimed to investigate the impact of haemoglobin levels on the outcomes of patients undergoing semi-urgent and elective PCI in an Asian population.

## Materials and Methods

This was a retrospective cohort database study of patients who underwent semi-urgent and elective PCI from January 2014 to December 2015 at a tertiary academic centre in Singapore. Patients who had emergency PCI for ST-segment elevation myocardial infarction were excluded. The patients were stratified into three groups based on their haemoglobin levels: normal haemoglobin level of ≥12 g/dL, an intermediate haemoglobin level of 10–11.9 g/dL, and a low haemoglobin level of <10 g/dL. The haemoglobin levels of the patients were taken on the admission of the visit when the procedure was to be done. The choice of the interventional device used during the PCI was at the discretion of the primary operator. The recommended duration of dual antiplatelet therapy (DAPT) given to the majority of the patients undergoing acute coronary syndrome was 1 year (13). The recommended duration following insertion of drug eluting stents, drug eluting balloons, bare metal stents, and plain old balloon angioplasty was 1 year, 6 months, 1 month, and 1 month, respectively (13, 14).

The patients’ demographic, anthropometric measurements, medical comorbidities, an indication of PCI, laboratory details, site of puncture, cardiac catheterisation findings, and the use of antiplatelet therapy and triple therapy (DAPT with Vitamin K antagonist or novel oral anticoagulant) after the procedure were recorded. The primary endpoint of the study was the composite of 5-point major adverse cardiac and cerebrovascular events (MACCE), defined as all-cause death, subsequent stroke/transient ischaemic attack, subsequent myocardial infarction, subsequent congestive cardiac failure, and target lesion revascularisation. The secondary endpoints of the study consisted of individual components of the primary endpoint, cardiovascular death, and also bleeding events. The definitions of individual components of all-cause death, cardiovascular death, subsequent stroke/transient ischaemic attack, subsequent myocardial infarction, subsequent congestive cardiac failure, and target lesion revascularisation were defined in accordance with the Standardized Data Collection for Cardiovascular Trials Initiative and the United States Food and Drug Administration (15). Subsequent stroke/transient ischaemic attack was inclusive of both early procedural stroke/transient ischaemic attack and the late-onset stroke/transient ischaemic attack. Early procedural stroke/transient ischaemic attack was defined as an event within 1 week of PCI, whereas late-onset stroke/transient ischaemic attack was defined as an event after 1 week of PCI. Bleeding events were defined as the development of bleeding events that were of Bleeding Academic Research Consortium type 3 and above (16). The endpoints were collected from both available clinic outpatient records and admission discharge summaries from the electronic medical records.

Categorical variables were reported with counts and percentages, and continuous variables were reported with the mean and 1 standard deviation. A comparison of demographic variables and pre-existing medical history between groups of patients with differing haemoglobin levels was made using the one-way ANOVA for continuous variables and Chi-square or Fisher’s exact tests as appropriate for categorical variables. The incidence of the primary composite endpoint of 5-point MACCE and secondary endpoints of all-cause death, cardiovascular death, subsequent stroke/transient ischaemic attack, subsequent myocardial infarction, subsequent congestive cardiac failure, and bleeding over a period of 3 years were studied using the Kaplan–Meier method and compared using the log-rank test. A multivariate Cox regression analysis including identified potential confounding demographic and clinical variables was carried out to identify independent predictors of the primary and secondary endpoint as well as for determination of the hazard ratios. The primary endpoint with 5-point MACCE was also separately analysed with the inclusion of only early procedural stroke/transient ischaemic attack instead of all subsequent stroke/transient ischaemic attacks. The potential confounders or variables that could contribute to morbidity and mortality after PCI such as age, gender, systolic blood pressure, smoking status, hypertension, hyperlipidaemia, diabetes mellitus, history of acute myocardial infarction, history of stroke/transient ischaemic attack, history of atrial fibrillation, history of congestive cardiac failure, history of chronic obstructive pulmonary disease/asthma, history of peripheral vascular disease, history of malignancy, history and severity of chronic kidney disease based on estimated glomerular filtration rate, previous PCI, previous coronary artery bypass graft surgery, type of PCI, choice of the puncture site, and left ventricular ejection fraction were included. Separate variables that could contribute to bleeding events post-PCI were included in the model for the analysis of bleeding events as endpoints. A two-tailed distribution was assumed for all *p*-values of <0.05 defined as being statistically significant. The analysis was performed using IBM SPSS Statistics Version 23 (IBM Corporation, Armonk, NY, United States).

Ethics approval was obtained from the local Institutional Review Board.

## Results

There were 1,685 patients available for analysis. The mean duration of follow-up for our patients in the study was 45 ± 29 months. Of the patients studied, there were 1,381 (82.0%) in the normal haemoglobin level group, 225 (13.4%) in the intermediate haemoglobin level group, and 79 (4.7%) in the low haemoglobin level group. The baseline demographics and pre-existing medication conditions are described in Table 1. Patients with low haemoglobin levels were predominantly male (54.4%) and Chinese (58.2%) with a mean age of 66.1 ± 13.3 years. Patients with intermediate and low haemoglobin levels also had a higher comorbid burden of advanced chronic kidney disease, atrial fibrillation, congestive cardiac failure, malignancy, and triple vessel disease compared to patients with normal haemoglobin levels. For patients with malignancy, the most common location of malignancy was the genitourinary tract tumour consisting of kidney, bladder, prostate malignancy with 31 (1.8%) patients (shown in Supplementary Table 1). Patients with intermediate and low haemoglobin levels were less likely to get radial puncture for their procedure, with 102 (45.3%) and 22 (27.8%), respectively, compared to patients with normal haemoglobin levels where 947 (68.6%) had a radial puncture. Patients with low haemoglobin levels were less likely to be offered ticagrelor and prasugrel as a choice of second anti-platelet agent in comparison to patients with normal haemoglobin levels.

**TABLE 1:** Baseline demographics.

Variable	Overall (*n* = 1685)	Hb ≥ 12 g/dL (*n* = 1381)	Hb 10–11.9 g/dL (*n* = 225)	Hb < 10 g/dL (*n* = 79)	*p-*value
Age (years)	61.1 ± 11.3	59.9 ± 10.9	66.7 ± 11.1^#^	66.1 ± 13.3^#^	**<0.001**
Female, *n* (%)	325 (19.3)	190 (13.8)	99 (44.0)^#^	36 (45.6)^#^	**<0.001**
Ethnicity					0.063
>Chinese, *n* (%)	1005 (59.6)	826 (59.8)	133 (59.1)	46 (58.2)	
>Malay, *n* (%)	275 (16.3)	213 (15.4)	47 (20.9)	15 (19.0)	
>Indian, *n* (%)	216 (12.8)	174 (12.6)	28 (12.4)	14 (17.7)	
>Others, *n* (%)	189 (11.2)	168 (12.2)	17 (7.6)	4 (5.1)	
Height (cm)	164.1 ± 8.5	165.2 ± 8.0	159.3 ± 9.0^#^	158.9 ± 9.7^#^	**<0.001**
Weight (kg)	70.6 ± 14.6	71.6 ± 14.2	65.2 ± 14.7^#^	68.0 ± 18.1	**<0.001**
BSA (m^2^)	1.79 ± 0.21	1.81 ± 0.21	1.70 ± 0.21^#^	1.74 ± 0.25	**<0.001**
Hb (g/dL)	13.7 ± 2.0	14.5 ± 1.3	11.1 ± 0.6^#^	9.1 ± 0.7^#@^	**<0.001**
Systolic blood pressure (mm Hg)	134 ± 25	132 ± 24	140 ± 25^#^	145 ± 26^#^	**<0.001**
Diastolic blood pressure (mm Hg)	75 ± 13	75 ± 13	70 ± 13^#^	70 ± 14^#^	**<0.001**
Smoking					**<0.001**
>Current smoker	498 (29.6)	453 (32.8)	37 (16.4)^#^	8 (10.1)^#^	
>Ex-smoker	269 (16.0)	222 (16.1)	34 (15.1)^#^	13 (16.5)^#^	
>Non-smoker	918 (54.5)	706 (51.1)	154 (68.4)^#^	58 (73.4)^#^	
Hypertension, *n* (%)	1193 (70.8)	927 (67.1)	194 (86.2)^#^	72 (91.1)^#^	**<0.001**
Dyslipidaemia, *n* (%)	1303 (77.3)	1052 (76.2)	186 (82.7)	65 (82.3)	0.055
Diabetes, *n* (%)	713 (42.3)	513 (37.1)	142 (63.1)^#^	58 (73.4)^#^	**<0.001**
History of ischaemic heart disease, *n* (%)	1671 (99.2)	1369 (99.1)	223 (99.1)	79 (100.0)	0.706
History of acute myocardial infarction, *n* (%)	1071 (63.6)	869 (62.9)	139 (61.8)	63 (79.7)^#@^	**0.009**
History of stroke or TIA, *n* (%)	140 (8.3)	99 (7.2)	30 (13.3)^#^	11 (13.9)^#^	**0.001**
History of atrial fibrillation, *n* (%)	116 (6.9)	67 (4.9)	30 (13.3)^#^	19 (24.1)^#@^	**<0.001**
History of congestive heart failure, *n* (%)	217 (12.9)	130 (9.4)	56 (24.9)^#^	31 (39.2)^#@^	**<0.001**
History of COPD/asthma, *n* (%)	114 (6.8)	92 (6.7)	17 (7.6)	5 (6.3)	0.874
History of peripheral vascular disease, *n* (%)	57 (3.4)	25 (1.8)	20 (8.9)^#^	12 (15.2)^#^	**<0.001**
History of malignancy, *n* (%)	126 (7.5)	85 (6.2)	27 (12.0)^#^	14 (17.7)^#^	**<0.001**
Chronic kidney disease (GFR categories)					**<0.001**
>Group 1 (G1)	685 (40.7)	633 (45.8)	42 (18.7)^#^	10 (12.7)^#@^	
>Group 2 (G2)	595 (35.3)	522 (37.8)	63 (28.0)^#^	10 (12.7)^#@^	
>Group 3 (G3)	222 (13.2)	153 (11.1)	52 (23.1)^#^	17 (21.5)^#@^	
>Group 3 (G4-5)	44 (2.6)	13 (0.9)	16 (7.1)^#^	15 (19.0)^#@^	
>Group 4 (Dialysis)	88 (5.2)	25 (1.8)	40 (17.8)^#^	23 (29.1)^#@^	
Indication of percutaneous coronary intervention					**0.014**
>NSTEMI, *n* (%)	943 (56.0)	754 (54.6)	131 (58.2)	58 (73.4)^#@^	
>Stable Angina, *n* (%)	537 (31.9)	452 (32.7)	70 (31.1)	15 (19.0)^#@^	
>Unstable Angina, *n* (%)	204 (12.1)	175 (12.7)	24 (10.7)	5 (6.3)^#@^	
Previous PCI, *n* (%)	515 (30.6)	408 (29.5)	81 (36.0)	26 (32.9)	0.134
Previous CABG, *n* (%)	114 (6.8)	77 (5.6)	29 (12.9)^#^	8 (10.1)	**<0.001**
Single vessel disease, *n* (%)	543 (32.2)	466 (33.7)	57 (25.3)^#^	20 (25.3)	**0.017**
Double vessel disease, *n* (%)	537 (31.9)	443 (32.1)	72 (32.0)	22 (27.8)	0.722
Triple vessel disease, *n* (%)	599 (35.5)	467 (33.8)	95 (42.2)^#^	37 (46.8)^#^	**0.005**
Type of PCI					**<0.001**
>2nd Generation DES, *n* (%)	1506 (89.4)	1250 (90.5)	194 (86.2)^#^	62 (78.5)^#^	
>BMS, *n* (%)	24 (1.4)	18 (1.3)	2 (0.9)^#^	4 (5.1)^#^	
>POBA, *n* (%)	44 (2.6)	28 (2.0)	13 (5.8)^#^	3 (3.8)^#^	
>DEB, *n* (%)	73 (4.3)	51 (3.7)	14 (6.2)^#^	8 (10.1)^#^	
>Thrombectomy, *n* (%)	4 (0.2)	3 (0.2)	1 (0.4)^#^	0 (0)^#^	
Anti-platelet therapy					0.060
>DAPT, *n* (%)	1537 (91.2)	1283 (92.9)	192 (85.3)	62 (78.5)	
>Triple Therapy, *n* (%)	60 (3.6)	45 (3.3)	9 (4.0)	6 (7.6)	
Choice of second anti-platelet					**<0.001**
>Clopidogrel, *n* (%)	1027 (60.9)	807 (58.4)	160 (71.1)^#^	60 (75.9)^#^	
>Ticagrelor, *n* (%)	502 (29.8)	456 (33.0)	39 (17.3)^#^	7 (8.9)^#^	
>Prasugrel, *n* (%)	86 (5.1)	78 (5.6)	7 (3.1)^#^	1 (1.3)^#^	
DAPT duration (months)	12 ± 3	12 ± 3	12 ± 4	12 ± 3	0.743
Duration of Triple Therapy (DAPT with Vitamin K antagonist or novel oral anticoagulant) (months)	3 ± 4	4 ± 4	3 ± 2	2 ± 1	0.303
Site of puncture (Radial), *n* (%)	1071 (63.6)	947 (68.6)	102 (45.3)^#^	22 (27.8)^#@^	**<0.001**
Left ventricular ejection fraction (%)	53 ± 13	54 ± 13	50 ± 15^#^	50 ± 13	**<0.001**
Contrast volume (ml)	139.1 ± 64.8	139.6 ± 65.4	134.4 ± 56.3	144.9 ± 75.0	0.394

*Values are expressed as mean ± SD or n (%).*

*BMS, bare metal stent; BSA, body surface area; CABG, coronary artery bypass graft surgery; COPD, chronic obstructive pulmonary disease; DEB; drug eluting balloon; DES, drug eluting stent; Hb, haemoglobin; NSTEMI, non-ST elevation myocardial infarction; PCI, percutaneous coronary intervention; POBA, plain old balloon angioplasty; TIA, transient ischaemic attack.*

*#Comparing with Hb < 12 g/dL.*

*@Comparing with Hb 10–11.9 g/dL.*

*The bold values are significant p-values of <0.05.*

**FIGURE 1:**
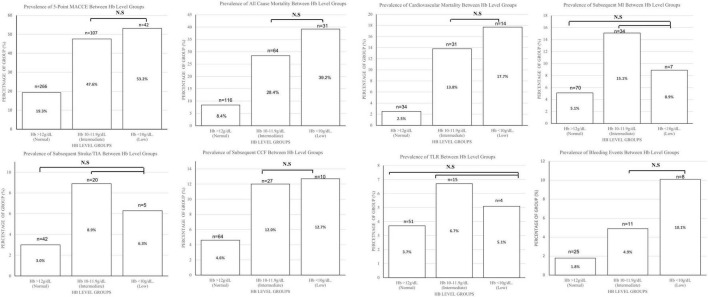
Charts comparing the 5-point MACCE, all-cause mortality, cardiovascular mortality, subsequent MI, subsequent Stroke/TIA, subsequent CCF, TLR, bleeding Events, and ≥BARC3 between haemoglobin level groups. Bar chart height shows the number of patients, while numbers in the bar charts show the percentage of patients that met the endpoint in each group. Comparisons between all groups are significant with *p* < 0.001 except for those stated as non-significant. BARC, bleeding academic research consortium; CCF, congestive cardiac failure; Hb, haemoglobin; MACCE, major adverse cardiac and cerebrovascular events; MI, myocardial infarction; N.S, non-significant; TIA, transient ischaemic attack; TLR, target lesion revascularisation.

**FIGURE 2:**
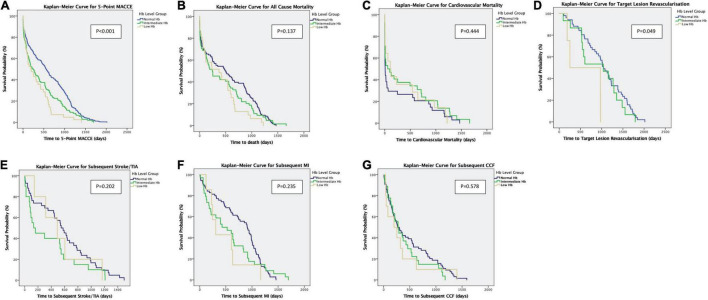
**(A–G)** Kaplan–Meier Curves show the primary outcomes of 5-point MACCE and secondary endpoints of all-cause mortality, cardiovascular mortality, subsequent stroke/TIA, subsequent MI, subsequent CCF, and target lesion revascularisation. CCF, congestive cardiac failure; TIA, transient ischaemic attack; MI, myocardial infarction; Hb, haemoglobin; MACCE, major adverse cardiac and cerebrovascular events.

In comparison to patients with normal haemoglobin levels, patients with low haemoglobin levels had a higher incidence of the primary endpoint of 5-point MACCE as well as secondary endpoints of all-cause death, cardiovascular death, subsequent stroke/transient ischaemic attack, subsequent myocardial infarction, subsequent congestive cardiac failure, and target lesion revascularisation as well as bleeding events (shown in Figure 1). Patients with low haemoglobin levels were associated with worse outcomes over the follow-up period (shown in Figure 2). Comparing patients with low haemoglobin levels to normal haemoglobin levels, the adjusted hazard ratio of 5-point MACCE was 1.89 (95% CI: 1.22, 2.92; *p* = 0.004) (shown in Table 2 and Supplementary Table 2). This was driven by the increased risk of target lesions revascularisation observed in the low haemoglobin level group compared to the normal haemoglobin level group (aHR 17.74, 95% CI: 1.74, 180.80; *p* = 0.015) (shown in Table 2 and Supplementary Table 3). A separate analysis of composite 5-point MACCE with the inclusion of early procedural stroke/transient ischaemic attack and exclusion of late-onset stroke/transient ischaemic attack also demonstrated that in comparison to patients with normal haemoglobin levels, patients with low haemoglobin levels were associated with a higher risk of 5-point MACCE with aHR of 1.75 (95% CI: 1.13, 2.71; *p* = 0.012) (shown in Supplementary Table 4). Patients with low haemoglobin levels had a higher association with bleeding events compared to patients with normal haemoglobin levels with an adjusted hazard ratio of 7.18, 95% CI: 1.13, 45.40; *p* = 0.036) (shown in Table 3).

**TABLE 2:** Unadjusted and adjusted hazard ratios of primary and secondary endpoints.

	Unadjusted Hazards Ratio (95% CI)			Adjusted Hazards Ratio (95% CI)^		
	Hb ≥ 12 g/dL (*n* = 1381)	Hb 10–11.9 g/dL (*n* = 225)	Hb < 10 g/dL (*n* = 79)	Hb ≥ 12 g/dL (*n* = 1381)	Hb 10–11.9 g/dL (*n* = 225)	Hb < 10 g/dL (*n* = 79)
**Primary endpoint**						
Composite of all-cause death, subsequent stroke/TIA, subsequent MI, subsequent CCF, target lesion revascularisation	Reference	1.26 (1.00, 1.59)	1.66 (1.18, 2.34)*	Reference	1.23 (0.90, 1.68)	1.89 (1.22, 2.92)*
**Secondary endpoint**						
All-cause death	Reference	1.18 (0.86, 1.60)	1.48 (0.99, 2.21)	Reference	1.04 (0.67, 1.62)	1.40 (0.79,2.46)
Cardiovascular death		0.74 (0.45, 1.21)	0.80 (0.43, 1.50)		0.54 (0.11, 2.67)	0.53 (0.20, 1.83)
Subsequent stroke/TIA		1.64 (0.95, 2.82)	1.18 (0.46, 3.00)		1.06 (0.38, 2.96)	0.21 (0.04, 1.18)
Subsequent MI		1.23 (0.80, 1.88)	1.85 (0.84, 4.05)		2.21 (0.90, 5.48)	3.02 (0.72, 12.67)
Subsequent CCF		1.26 (0.80, 1.99)	1.20 (0.61, 2.38)		1.05 (0.53, 2.07)	2.27 (0.62, 8.35)
Target Lesion Revascularisation		1.28 (0.71, 2.28)	3.42 (1.19, 9.84)*		0.87 (0.25, 3.02)	17.74 (1.74, 180.80)*

*CCF, congestive cardiac failure; CI, confidence interval; Hb, haemoglobin; MI, myocardial infarction; TIA, transient ischaemic attack.*

*p < 0.05.

*^Represents adjustment for confounders including age, gender, systolic blood pressure, smoking status, hypertension, hyperlipidaemia, diabetes mellitus, history of acute myocardial infarction, history of stroke/TIA, history of atrial fibrillation, history of CCF, history of chronic obstructive pulmonary disease/asthma, history of peripheral vascular disease, history of malignancy, history and severity of chronic kidney disease based on estimated glomerular filtration rate, previous percutaneous coronary intervention, previous coronary artery bypass graft surgery, type of percutaneous coronary intervention, puncture site (radial, femoral), left ventricular ejection fraction.*

**TABLE 3:** Cox regression analysis of predictors of significant bleeding events (Bleeding Academic Research Consortium Type 3 or worse).

Variables	Univariate analysis			Multivariate analysis		
	HR	95% CI	*p*-value	HR	95% CI	*p*-value
Age	1.01	1.00–1.04	0.289	1.01	0.97–1.05	0.618
**Smoking**						
Non-smoker	Reference	1		Reference	1	
Ex-smoker	0.78	0.39–1.58	0.498	0.99	0.38–2.63	0.989
Current smoker	1.11	0.64–1.92	0.705	1.53	0.65–3.64	0.331
**Site of puncture**						
Radial	Reference	1	**0.025**	Reference	1	0.095
Femoral	1.82	1.08–3.08		2.01	0.89–4.55	
**2nd antiplatelet**						
Clopidogrel	Reference	1		Reference	1	
Ticagrelor	1.10	0.63–1.91	0.747	1.18	0.45–3.10	0.740
Prasugrel	0.57	0.14–2.39	0.441	0.71	0.12–4.13	0.707
**Anti-platelet therapy**						
DAPT	Reference	1		Reference		
Triple Therapy (DAPT with Vitamin K antagonist or novel oral anticoagulant)	1.80	0.56–5.82	0.326	2.22	0.30–16.51	0.436
**Chronic kidney disease (GFR categories)**						
Group 1 (G1)	Reference	1		Reference	1	
Group 2 (G2)	1.39	0.76–2.54	0.291	1.71	0.68–4.28	0.256
Group 3 (G3)	1.68	0.76–3.69	0.198	2.34	0.53–10.37	0.264
Group 3 (G4-5)	2.56	0.91–7.18	0.075	2.20	0.45–10.73	0.331
Group 4 (Dialysis)	2.95	1.11–7.83	**0.030**	0.95	0.14–6.49	0.957
**Haemoglobin**						
−Hb > 12	Reference	1		Reference	1	
−Hb 10−11.9	1.15	0.61−2.20	0.664	0.87	0.25−3.01	0.830
−Hb < 10	2.26	1.03−4.94	**0.041**	7.18	1.13−45.40	**0.036**
Hypertension	0.73	0.40−1.33	0.298	0.65	0.25−1.75	0.397
Diabetes Mellitus	1.30	0.79−2.13	0.299	1.82	0.75−4.41	0.188
Dyslipidaemia	1.75	1.01−3.01	**0.044**	2.49	0.97−6.38	0.057
History of Atrial Fibrillation	0.95	0.45−2.00	0.900	3.42	0.77−15.18	0.106
History of Congestive Cardiac Failure	0.55	0.28−1.07	0.079	0.91	0.28−2.95	0.868
History of Stroke/Transient Ischaemic Attack	0.47	0.20−1.10	0.083	0.40	0.08−2.02	0.270
History of COPD/asthma	1.04	0.38−2.87	0.945	1.71	0.32−9.29	0.535
History of Peripheral Vascular Disease	0.33	0.05−2.49	0.284	0.54	0.02−14.84	0.713
History of Acute Myocardial Infarction	0.90	0.52−1.55	0.697	0.98	0.39−2.43	0.964

*CI, confidence interval; COPD, chronic obstructive pulmonary disease; DAPT; dual antiplatelet therapy, GFR; glomerular filtration rate, Hb; haemoglobin, HR, hazards ratio. The bold values are significant p-values of <0.05.*

Patients in the intermediate haemoglobin level group did not have significantly worse outcomes when compared to the normal haemoglobin level group. Comparing patients with intermediate haemoglobin levels to normal haemoglobin levels, the adjusted hazard ratio of 5-point MACCE was 1.23 (95% CI: 0.90, 1.68; *p* = 0.201) (shown in Table 2). The adjusted hazard ratio of bleeding events comparing patients with intermediate haemoglobin levels to normal haemoglobin levels was 0.87 (95% CI: 0.25, 3.01; *p* = 0.830) (shown in Table 3).

## Discussion

In our study of 1,685 patients from an Asian PCI cohort, patients with low haemoglobin levels were associated with poorer long-term outcomes after PCI. However, the periprocedural outcomes for a patient with intermediate haemoglobin levels were not significantly different when compared to patients with normal haemoglobin levels.

Patients with low haemoglobin levels often have other concomitant cardiovascular risk factors which may contribute to worse cardiac mortality and morbidity. In a retrospective study by Jiang et al. there was a suggestion that low haemoglobin levels pre-PCI was not an independent predictor of any clinical event post-PCI, after taking into account other comorbidities as confounders (17). Similarly, in our study, the patients had similar demographics throughout the spectrum of low and intermediate haemoglobin level groups: being older and predominantly male. In our study, patients with low haemoglobin levels had a higher comorbid burden. However, despite adjustment for comorbidities that might be potential confounders, low haemoglobin levels continued to be an independent predictor of 5-point MACCE risk compared to those with normal or intermediate haemoglobin levels, as opposed to the results reported by Jiang et al. Notably, our study differed in the longer follow-up period of 3.8 years compared to their follow-up period of 2 years in the study.

Significantly, our study population consists of a high prevalence of patients with chronic kidney disease, where anaemia is a known complication. Prior studies have elected to exclude patients with advanced chronic kidney in the analysis of outcomes post-PCI in patients with low haemoglobin levels (4, 6). In our study, despite accounting for chronic kidney disease, patients with low haemoglobin levels were more likely to have worse outcomes compared to those without. These results are consistent with the study by Kitai et al. where low haemoglobin levels were an independent risk factor for poorer outcomes post-PCI even after adjustment for chronic kidney disease (18). Our results further extend the understanding of the impact of differing levels of haemoglobin on 5-point MACCE, including an additional endpoint of subsequent stroke/transient ischaemic attack post-PCI. For cerebrovascular events after both emergency and elective PCI, it has been well established that stroke/transient ischaemic attack complicating PCI is a rare but serious event post-PCI with an estimated incidence of 0.4% of all PCI procedures (19, 20). However, there has been no established consensus on the topic of subsequent stroke post-PCI in terms of the causal relationship between PCI and the timing of the event post-PCI, with some studies demonstrating that subsequent stroke after PCI happened early, with the highest risk within a week post-procedure, while other studies associated delayed or late-onset stroke happening months to years after as a non-negligible complication related to PCI as well (21, 22). Apart from the 5-point MACCE, including all subsequent strokes/transient ischaemic attack, our study attempted to evaluate the impact of low haemoglobin level on composite adverse events with isolated early procedural stroke/transient ischaemic attack post-PCI, and our findings demonstrated that low haemoglobin level was also associated with an increased risk of 5-point MACCE (with isolated early procedural stroke). Given the rarity and severity of cerebrovascular complications post-PCI, more studies would be required to further evaluate the temporal relationship between PCI and subsequent stroke/transient ischaemic attack.

Patients with malignancy were more inclined to have more medical comorbidities such as hypertension, worse renal function, and lower haemoglobin levels compared to patients without malignancy (23–25). In our study, the patients with low haemoglobin levels were more likely to have a past medical history of malignancy. Patients with malignancy may develop low haemoglobin levels due to multiple factors, such as cytokine-mediation resulting from complex interactions between the tumour cell and the immune system apart from traditional causes like bone marrow infiltration, blood loss, haemolysis, renal causes, or chemotherapy-related causes (26). Some studies have demonstrated the importance of malignancy as a predictor of poor outcome post-PCI (25, 27). However, prior studies on the association between low haemoglobin levels and post-PCI outcomes did not include analysis of malignancy as a past medical history as a potential confounder of poor PCI outcomes (5, 6, 17). Interestingly, in our study, despite accounting for malignancy, patients with low haemoglobin levels remained as an independent predictor of worse post-PCI outcomes compared to those without.

Coronary artery disease in patients with low haemoglobin levels may manifest differently compared to patients with normal haemoglobin levels. Patients with low haemoglobin levels typically have non-atheromatous vascular remodelling of the coronary arteries as a result of long-standing increased haemodynamic load and pressure (28). However, low haemoglobin levels are also associated with increased inflammatory biomarkers such as C-reactive protein, which is suggestive of ongoing inflammation (29, 30). Consequently, these patients are at a higher risk of atherosclerosis and increased coronary plaque burden (31, 32). They would theoretically exhibit more complex, diffused, heavily calcified arteries (including coronary arteries with multiple coronary vessel involvement) during a coronary angiogram, in particular, if comorbidities include chronic kidney disease or diabetes mellitus (33). In our study, there was a higher proportion of patients with triple vessel disease in patients with low haemoglobin levels compared to those with intermediate haemoglobin levels. The complex coronary anatomy increased the difficulties of PCI in terms of stent deployment and achievement of optimal revascularisation. Complex coronary morphology may explain why patients with low haemoglobin levels have inferior immediate and long-term outcomes on subsequent follow-up (34). Furthermore, in our study, we noted that despite low haemoglobin levels, more patients underwent PCI *via* transfemoral access compared to transradial access. As the choice of access is decided by the operator, the choice of transfemoral access for these groups of patients may be secondary to the complex coronary morphology with a greater need for easier catheter manipulation. However, since the MATRIX study, there has been increasing evidence from recent studies that transradial access is associated with lower rates of major adverse cardiovascular events and complications including bleeding risk and reduced length of hospital stay with improved patient comfort compared to transfemoral PCI access (35–40). This is increasingly recognised with recent consensus and guidelines, with the choice of transradial recommended over transfemoral access (41, 42). Our finding highlights a gap to raise awareness on the benefits of transradial access and its lower complications and risks amongst clinicians.

Bleeding complications post-PCI is one of the most common complications and contributes to morbidity and mortality post-procedure (43, 44). Studies have shown that low haemoglobin level is an independent risk factor for bleeding complications in acute coronary syndromes (6, 45–47). Postulated reasons for increased risk of bleeding in patients with low haemoglobin levels were that the low haemoglobin level could be due to occult bleeding from the gastrointestinal tract or malignancy, inflammation, or bleeding diathesis, which might be tipped over by the usage of DAPT post-PCI (46). Comparably, our study also demonstrated that patients with low haemoglobin levels were more likely to develop bleeding events post-PCI as compared to patients with normal haemoglobin levels. It is well described in the “East Asian Paradox” that Asians are more likely to develop bleeding complications post-PCI compared to Caucasians; therefore clinicians are more cautious in the choice of antiplatelet therapy (48). In comparison with patients with normal haemoglobin levels, our study population with low haemoglobin levels was less likely to be offered ticagrelor or prasugrel as the choice of second antiplatelet agent, which was more effective but conferred a higher risk of bleeding when compared to clopidogrel (49, 50). Interestingly, despite the adjustment for thrombotic or anticoagulation therapies that predispose to increased risk of bleeding as well as other medical comorbidities, a low haemoglobin level remained independently associated with increased bleeding events. Similar to previous studies, our study further adds to the knowledge that a low haemoglobin level of below 10 g/dl is an independent predictor of major bleeding events of BARC3 or worse in patients undergoing semi-urgent and elective PCI. However, compared to the study by Manoukian et al., Voeltz et al., and Bassand et al. patients in the intermediate haemoglobin level group of haemoglobin 10–11.9 g/dl did not have a higher association with bleeding complications post-procedure. Another difference between our study and prior studies is the exclusion of STEMI patients in our study (6, 45–47). With increased major bleeding complications during the procedure and after in the immediate- and long-term, these subsets of patients are more likely to have a poorer outcome post-PCI (45, 51).

Dual antiplatelet therapy is a crucial anti-thrombotic therapy for acute coronary syndromes and reduces the risk of in-stent thrombosis post-PCI. With regards to the choice of the second antiplatelet agent, there has been extensive debate regarding its choice in terms of efficacy as well as safety or bleeding profiles amongst ticagrelor, prasugrel, and clopidogrel. In the earlier days, when compared to clopidogrel, prasugrel was noted to have a higher risk of major bleeding in the TRITON-TIMI 38 study, and ticagrelor was observed to have an increase in non-procedure related bleeding in the PLATO study (49, 50, 52). However, recent studies suggested that there is variability to the bleeding profiles and that the three antiplatelet agents may be equivalent in their bleeding profiles (53–56). In our study of patients undergoing semi-urgent or elective PCI, similar to recent studies by Krishnamurthy et al. and meta-analysis by Baldetti et al. there are no significant differences in major bleeding outcomes between clopidogrel, prasugrel, and ticagrelor (53, 54).

In patients assessed to be at higher risk of bleeding, international society recommendations, such as the European Society of Cardiology guidelines on myocardial revascularisation, recommend a shortened DAPT duration of 1 month for the usage of new generation drug-eluting stents (57). These new generation drug-eluting stents have also shown safety in shortened DAPT duration in the recent LEADERS FREE trial and ONYX-One trial (58, 59). Currently, there is no clear evidence supporting the role of periprocedural blood transfusion for patients with low haemoglobin levels. A recent systemic review and meta-analysis had shown that blood transfusion was independently associated with increased cardiovascular events in acute myocardial infarction and PCI (60). Furthermore, in the CRIT randomised pilot trial, treatment of anaemic patients with acute myocardial infarction with blood transfusion to maintain a higher haematocrit was associated with a poorer clinical outcome of death, recurrent myocardial infarction, and congestive cardiac failure (61). However, some observational analyses have reported that the worse outcomes in this group of patients may be confounded by the severity of acute myocardial infarction (62, 63). Hence, to the best of our knowledge, there is currently no clear and definite guideline regarding blood transfusion prior to PCI in this population.

There have been debates regarding the definition of anaemia as well as the normal ranges of haemoglobin for different populations. Studies have shown that laboratory reference intervals for commonly measured analytes can differ between different populations, including haemoglobin levels (64–66). A large national database in the United States, US National Health and Nutrition Examination Survey (NHANES) 2011–2012 showed that the normal laboratory range of haemoglobin level Asian Population significantly shifted to lower levels compared to the Caucasian population (65). With these uncertainties regarding the low normal reference value of haemoglobin levels and the definition of anaemia in Asian patients, it is imperative to further study the gaps in the impact of anaemia on Asian populations undergoing non-urgent, elective PCI.

As the exact level of haemoglobin that affects outcome is yet to be determined, our observational study on the Asian population hopes to add to the literature to evaluate the severity of low haemoglobin, which is of significant clinical importance, that would predict for worse MACCE after non-urgent, elective PCI. This would allow clinicians to evaluate the benefit and risk for patients with low haemoglobin levels undergoing PCI, to put in place measures to reduce the risk of major bleeding in anaemic patients. Our study demonstrated the associated poor outcomes due to low haemoglobin levels. As such, we recommend the consideration of optimal medical therapy prior to PCI in these high risk groups of patients. PCI may be considered if there is a failure of optimal medical therapy or severe complications of coronary artery disease if treated conservatively, such as a large area of inducible ischaemia and/or left ventricular dysfunction.

Our study’s strength lies in the utilisation of a real-world registry in the evaluation of Asian patients with low haemoglobin levels undergoing PCI in a semi-urgent and elective setting. The duration of follow-up in our study was long enough to detect outcomes up to 4 years. However, we also acknowledge the limitations of our study. First, we were unable to evaluate the duration and provision of guideline-directed medical therapy such as angiotensin II converting enzyme inhibitors, angiotensin II receptor blockers used, as well as the cause of low haemoglobin level since it was not available in this database. Second, the retrospective nature of our study design leaves it susceptible to selection bias and loss to follow-up. Third, we were limited to examining associations and not causation due to the observational nature of our study.

## Conclusion

Patients with low haemoglobin levels who underwent semi-urgent and elective PCI had a higher comorbidity burden and were associated with worse outcomes on long-term follow-up. More studies are required to further evaluate the risks and benefits of PCI to allow clinicians to make clinically appropriate decisions for the management of stable coronary artery disease in patients with low haemoglobin levels.

## Data Availability Statement

The datasets presented in this article are not readily available because data will require appropriate institutional clearance and permission. Requests to access the datasets should be directed to the corresponding author (CH-S), ching_hui_sia@nuhs.edu.sg.

## Ethics Statement

The studies involving human participants were reviewed and approved by the National Healthcare Group Domain Specific Review Board (NHG-DSRB). Written informed consent for participation was not required for this study in accordance with the national legislation and the institutional requirements.

## Author Contributions

RY-HS and C-HS were involved in the majority of the manuscript from the conception and design, analysis and interpretation of data, and drafting of the manuscript and revision. R-HL, P-YH, JW-HN, and JS-YH were involved in the conception and design, and analysis and interpretation of data. AD and H-WS were involved in the drafting of the manuscript and revision. T-CY, H-CT, MY-YC, and JP-YL were involved in the drafting of the manuscript, revision, and giving the final approval of the manuscript submitted. All authors contributed to the article and approved the submitted version.

## Conflict of Interest

The authors declare that the research was conducted in the absence of any commercial or financial relationships that could be construed as a potential conflict of interest.

## Publisher’s Note

All claims expressed in this article are solely those of the authors and do not necessarily represent those of their affiliated organizations, or those of the publisher, the editors and the reviewers. Any product that may be evaluated in this article, or claim that may be made by its manufacturer, is not guaranteed or endorsed by the publisher.
