# Supplementary material for: Clinical Characteristics and Long-Term Outcomes of Patients With Differing Haemoglobin Levels Undergoing Semi-Urgent and Elective Percutaneous Coronary Intervention in an Asian Population
Source: Front Cardiovasc Med. 2022 Mar 18;9:687555. doi: 10.3389/fcvm.2022.687555 (PMC8971291; doi:10.3389/fcvm.2022.687555)
Supplement: Supplementary file 1 [file Data_Sheet_1.docx]

**Supplementary Table 1. Location/Type of Malignancy**

| **Location/Type of Malignancy** | **No. of Patients with Malignancy**  **(n=126)** |
| --- | --- |
| Genitourinary Tract (kidney, bladder, prostate) | 31 (1.8) |
| Colorectal | 16 (0.9) |
| Breast | 13 (0.8) |
| Lung (including pleural) | 13 (0.8) |
| Liver | 11 (0.7) |
| Head, ear, nose, throat (including thyroid) | 11 (0.7) |
| Gastrointestinal (excluding colorectal, liver) | 10 (0.6) |
| Gynaecological (Cervical, Endometrial, Ovarian) | 9 (0.5) |
| Haematological | 8 (0.5) |
| Skin | 3 (0.2) |
| Central Nervous System | 1 (0.1) |

**Supplemental Table 2. Cox regression analysis of predictors of 5-Point Major Adverse Cardiac and Cerebrovascular Events with Target Lesion Revascularisation**

Abbreviations: BMS, bare metal stent; CABG, coronary artery bypass graft surgery; CI; confidence interval; COPD, chronic obstructive pulmonary disease; DEB; drug eluting balloon; DES, drug eluting stent; GFR, glomerular filtration rate; HR, hazards ratio; PCI, percutaneous coronary intervention; POBA, plain old balloon angioplasty; TIA, transient ischaemic attack.

| **Variables** | **Univariate Analysis** | | | **Multivariate Analysis** | | |
| --- | --- | --- | --- | --- | --- | --- |
|  | **HR** | **95% CI** | **p value** | **HR** | **95% CI** | **p value** |
| Age | 1.02 | 1.01-1.03 | **<0.001** | 1.02 | 1.00-1.03 | **0.009** |
| Female | 0.82 | 0.66-1.03 | 0.086 | 0.97 | 0.71-1.33 | 0.856 |
| Ethnicity  Chinese  Malay  Indian  Others | Reference  1.05  0.92  0.94 | 1  0.81-1.36  0.71-1.18  0.64-1.36 | 0.731  0.506  0.728 |  |  |  |
| Systolic blood pressure | 1.00 | 0.99-1.00 | 0.153 | 1.00 | 1.00-1.00 | 0.824 |
| Smoking  Non-smoker  Ex-smoker  Current smoker | Reference  0.96  0.83 | 1  0.73-1.26  0.66-1.04 | 0.764  0.108 | 1.04  1.06 | 0.73-1.47  0.78-1.45 | 0.833  0.706 |
| Hypertension | 0.94 | 0.73-1.21 | 0.628 | 0.84 | 0.60-1.16 | 0.289 |
| Dyslipidaemia | 1.57 | 1.23-2.02 | **<0.001** | 1.66 | 1.19-2.32 | **0.003** |
| Diabetes | 0.88 | 0.72-1.07 | 0.210 | 0.92 | 0.70-1.20 | 0.538 |
| History of acute myocardial infarction | 0.78 | 0.61-0.99 | **0.038** | 0.86 | 0.64-1.16 | 0.314 |
| History of stroke or TIA | 1.12 | 0.85-1.47 | 0.433 | 1.12 | 0.80-1.56 | 0.513 |
| History of atrial fibrillation | 0.72 | 0.55-0.95 | **0.018** | 0.98 | 0.69-1.40 | 0.923 |
| History of congestive heart failure | 0.74 | 0.60-0.91 | **0.005** | 0.91 | 0.67-1.21 | 0.504 |
| History of COPD / asthma | 1.27 | 0.89-1.82 | 0.189 | 1.34 | 0.89-2.01 | 0.166 |
| History of peripheral vascular disease | 0.79 | 0.56-1.11 | 0.173 | 1.00 | 0.64-1.57 | 0.997 |
| History of malignancy | 1.01 | 0.77-1.32 | 0.968 | 1.29 | 0.92-1.82 | 0.146 |
| Chronic kidney disease (GFR categories)  Group 1 (G1)  Group 2 (G2)  Group 3 (G3)  Group 3 (G4-5)  Group 4 (Dialysis) | Reference  1.12  1.40  2.10  1.12 | 1  0.85-1.46  1.05-1.85  1.35-3.25  0.81-1.56 | 0.418  **0.020**  0.001  0.483 | Reference  0.99  0.79  1.14  0.69 | 1  0.73-1.36  0.54-1.16  0.60-2.14  0.44-1.09 | 0.973  0.233  0.691  0.110 |
| Previous PCI | 1.08 | 0.88-1.32 | 0.458 | 1.12 | 0.88-1.43 | 0.366 |
| Previous CABG | 0.90 | 0.67-1.21 | 0.485 | 0.99 | 0.70-1.41 | 0.969 |
| Choice of second anti-platelet  Clopidogrel  Ticagrelor  Prasugrel | Reference  1.16  1.20 | 0.92-1.48  0.71-2.03 | 0.211  0.488 |  |  |  |
| Haemoglobin  Hb> 12  Hb 10-11.9  Hb<10 | Reference  1.43  1.92 | 1  1.14-1.79  1.38-2.68 | **0.002**  **<0.001** | Reference  1.23  1.89 | 1  0.90-1.68  1.22-2.92 | 0.201  **0.004** |
| Type of PCI  2^nd^ Generation DES  BMS  POBA  DEB  Thrombectomy | Reference  1.92  1.28  1.06  1.56 | 1  1.14-3.23  0.81-2.01  0.71-1.58  0.39-6.28 | **0.014**  0.292  0.778  0.530 | Reference  1.22  2.95  0.94  0.79 | 1  0.64-2.34  1.59-5.48  0.60-1.48  0.18-3.53 | 0.547  **0.001**  0.792  0.761 |
| Site of puncture  Radial  Femoral | Reference  1.27 | 1  1.04-1.54 | **0.020** | Reference  1.23 | 1  0.96-1.59 | 0.106 |
| Left ventricular ejection fraction | 0.99 | 0.98-0.99 | **<0.001** | 0.99 | 0.98-1.00 | **0.006** |

**Supplemental Table 3. Cox regression analysis of predictors of Target Lesion Revascularisation**

Abbreviations: CABG, coronary artery bypass graft surgery; CI; confidence interval; COPD, chronic obstructive pulmonary disease; GFR, glomerular filtration rate; HR, hazards ratio; PCI, percutaneous coronary intervention; TIA, transient ischaemic attack.

| **Variables** | **Univariate Analysis** | | | **Multivariate Analysis** | | |
| --- | --- | --- | --- | --- | --- | --- |
|  | **HR** | **95% CI** | **p value** | **HR** | **95% CI** | **p value** |
| Age | 0.98 | 0.96-1.00 | 0.104 | 0.98 | 0.94-1.02 | 0.233 |
| Female | 0.87 | 0.45-1.67 | 0.677 | 0.84 | 0.24-3.00 | 0.793 |
| Ethnicity  Chinese  Malay  Indian  Others | Reference  1.23  1.33  0.93 | 1  0.58-2.60  0.75-2.36  0.36-2.39 | 0.595  0.338  0.885 |  |  |  |
| Systolic blood pressure | 1.00 | 0.99-1.01 | 0.903 | 1.00 | 0.98-1.01 | 0.856 |
| Smoking  Non-smoker  Ex-smoker  Current smoker | Reference  1.05  0.69 | 1  0.53-2.07  0.41-1.17 | 0.886  0.170 | 1.31  0.68 | 0.42-4.09  0.28-1.66 | 0.648  0.400 |
| Hypertension | 1.02 | 0.57-1.81 | 0.957 | 0.44 | 0.17-1.16 | 0.098 |
| Dyslipidaemia | 1.61 | 0.73-3.54 | 0.236 | 0.73 | 0.18-3.03 | 0.669 |
| Diabetes | 0.96 | 0.59-1.57 | 0.871 | 1.93 | 0.79-4.67 | 0.148 |
| History of acute myocardial infarction | 1.24 | 0.75-2.05 | 0.404 | 1.54 | 0.61-3.86 | 0.358 |
| History of stroke or TIA | 1.30 | 0.66-2.55 | 0.452 | 0.73 | 0.27-1.97 | 0.537 |
| History of atrial fibrillation | 0.15 | 0.04-0.68 | **0.014** | 0.05 | 0.00-0.59 | **0.018** |
| History of congestive heart failure | 1.60 | 0.79-3.24 | 0.196 | 7.34 | 1.77-30.46 | **0.006** |
| History of COPD / asthma | 1.48 | 0.59-3.75 | 0.406 | 1.60 | 0.51-5.05 | 0.421 |
| History of peripheral vascular disease | 0.65 | 0.24-1.81 | 0.414 | 0.22 | 0.03-1.49 | 0.121 |
| History of malignancy | 0.97 | 0.44-2.14 | 0.940 | 2.31 | 0.64-8.30 | 0.201 |
| Chronic kidney disease (GFR categories)  Group 1 (G1)  Group 2 (G2)  Group 3 (G3)  Group 3 (G4-5)  Group 4 (Dialysis) | Reference  0.96  0.64  1.13  2.19 | 1  0.54-1.71  0.29-1.41  0.27-4.78  1.04-4.61 | 0.889  0.271  0.871  **0.040** | Reference  0.57  1.45  2.47  0.33 | 1  0.23-1.42  0.41-5.16  0.20-31.07  0.05-2.11 | 0.230  0.570  0.483  0.241 |
| Previous PCI | 0.78 | 0.48-1.27 | 0.312 | 0.62 | 0.28-1.38 | 0.241 |
| Previous CABG | 1.37 | 0.70-2.68 | 0.362 | 3.11 | 0.86-11.22 | 0.083 |
| Choice of second anti-platelet  Clopidogrel  Ticagrelor  Prasugrel | Reference  1.35  1.88 | 0.76-2.37  0.73-4.84 | 0.305  0.189 |  |  |  |
| Haemoglobin  Hb> 12  Hb 10-11.9  Hb<10 | Reference  1.28  3.42 | 1  0.71-2.28  1.18-9.84 | 0.414  **0.023** | Reference  0.87  17.74 | 1  0.25-3.02  1.74-180.80 | 0.822  **0.015** |
| Site of puncture  Radial  Femoral | Reference  1.32 | 1  0.81-2.13 | 0.264 | Reference  1.28 | 1  0.61-2.67 | 0.511 |
| Left ventricular ejection fraction | 1.01 | 0.98-1.03 | 0.601 | 0.98 | 0.94-1.03 | 0.418 |

**Supplemental Table 4. Cox regression analysis of predictors of 5-Point Major Adverse Cardiac and Cerebrovascular Events with Target Lesion Revascularisation (For subsequent stroke/TIA component within 1 week of PCI)**

Abbreviations: BMS, bare metal stent; CABG, coronary artery bypass graft surgery; CI; confidence interval; COPD, chronic obstructive pulmonary disease; DEB; drug eluting balloon; DES, drug eluting stent; GFR, glomerular filtration rate; HR, hazards ratio; PCI, percutaneous coronary intervention; POBA, plain old balloon angioplasty; TIA, transient ischaemic attack.

| **Variables** | **Univariate Analysis** | | | **Multivariate Analysis** | | |
| --- | --- | --- | --- | --- | --- | --- |
|  | **HR** | **95% CI** | **p value** | **HR** | **95% CI** | **p value** |
| Age | 1.02 | 1.01-1.03 | **<0.001** | 1.02 | 1.00-1.03 | **0.014** |
| Female | 0.83 | 0.66-1.05 | 0.113 | 1.04 | 0.75-1.45 | 0.824 |
| Ethnicity  Chinese  Malay  Indian  Others | Reference  1.13  0.92  1.02 | 1  0.86-1.48  0.71-1.19  0.68-1.52 | 0.367  0.512  0.936 |  |  |  |
| Systolic blood pressure | 1.00 | 0.99-1.00 | 0.063 | 1.00 | 1.00-1.00 | 0.645 |
| Smoking  Non-smoker  Ex-smoker  Current smoker | Reference  0.96  0.77 | 1  0.72-1.27  0.61-0.97 | 0.766  0.028 | 1.05  0.95 | 0.73-1.52  0.68-1.33 | 0.799  0.759 |
| Hypertension | 1.02 | 0.78-1.32 | 0.909 | 0.84 | 0.59-1.19 | 0.320 |
| Dyslipidaemia | 1.62 | 1.25-2.10 | **<0.001** | 1.69 | 1.19-2.40 | **0.004** |
| Diabetes | 0.92 | 0.75-1.13 | 0.417 | 0.95 | 0.71-1.28 | 0.753 |
| History of acute myocardial infarction | 0.79 | 0.61-1.01 | 0.055 | 0.86 | 0.63-1.18 | 0.355 |
| History of stroke or TIA | 1.24 | 0.93-1.65 | 0.153 | 1.16 | 0.82-1.63 | 0.414 |
| History of atrial fibrillation | 0.68 | 0.52-0.91 | **0.008** | 0.94 | 0.65-1.36 | 0.743 |
| History of congestive heart failure | 0.74 | 0.60-0.92 | **0.007** | 0.96 | 0.71-1.30 | 0.801 |
| History of COPD / asthma | 1.35 | 0.93-1.95 | 0.115 | 1.29 | 0.84-1.97 | 0.245 |
| History of peripheral vascular disease | 0.84 | 0.59-1.20 | 0.341 | 1.13 | 0.71-1.79 | 0.600 |
| History of malignancy | 1.03 | 0.78-1.37 | 0.834 | 1.36 | 0.95-1.94 | 0.960 |
| Chronic kidney disease (GFR categories)  Group 1 (G1)  Group 2 (G2)  Group 3 (G3)  Group 3 (G4-5)  Group 4 (Dialysis) | Reference  1.13  1.46  2.37  1.15 | 1  0.85-1.50  1.09-1.96  1.49-3.77  0.82-1.62 | 0.402  **0.012**  **<0.001**  0.415 | Reference  1.01  0.87  1.27  0.75 | 1  0.72-1.41  0.58-1.31  0.62-2.59  0.47-1.20 | 0.960  0.509  0.516  0.224 |
| Previous PCI | 1.10 | 0.89-1.35 | 0.390 | 1.15 | 0.89-1.48 | 0.296 |
| Previous CABG | 1.02 | 0.75-1.38 | 0.898 | 1.21 | 0.83-1.75 | 0.325 |
| Choice of second anti-platelet  Clopidogrel  Ticagrelor  Prasugrel | Reference  1.24  1.33 | 0.96-1.59  0.77-2.29 | 0.100  0.305 |  |  |  |
| Haemoglobin  Hb> 12  Hb 10-11.9  Hb<10 | Reference  1.35  1.80 | 1  1.07-1.71  1.29-2.51 | **0.012**  **0.001** | Reference  1.18  1.75 | 1  0.85-1.64  1.13-2.71 | 0.333  **0.012** |
| Type of PCI  2^nd^ Generation DES  BMS  POBA  DEB  Thrombectomy | Reference  2.04  1.25  1.10  1.67 | 1  1.16-3.56  0.78-1.98  0.73-1.64  0.42-6.73 | **0.013**  0.355  0.653  0.470 | Reference  1.15  2.39  1.01  0.88 | 1  0.56-2.37  1.28-4.47  0.64-1.58  0.20-3.93 | 0.707  **0.006**  0.973  0.863 |
| Site of puncture  Radial  Femoral | Reference  1.22 | 1  0.99-1.50 | 0.062 | Reference  1.14 | 1  0.88-1.50 | 0.326 |
| Left ventricular ejection fraction | 0.99 | 0.98-0.99 | **<0.001** | 0.98 | 0.98-0.99 | **0.001** |
